# Supplementary figures and images for: A role for mitochondria–ER crosstalk in amyotrophic lateral sclerosis 8 pathogenesis
Source: Life Sci Alliance. 2025 Jan 27;8(4):e202402907. doi: 10.26508/lsa.202402907 (PMC11772500; doi:10.26508/lsa.202402907)

Fig. 2A

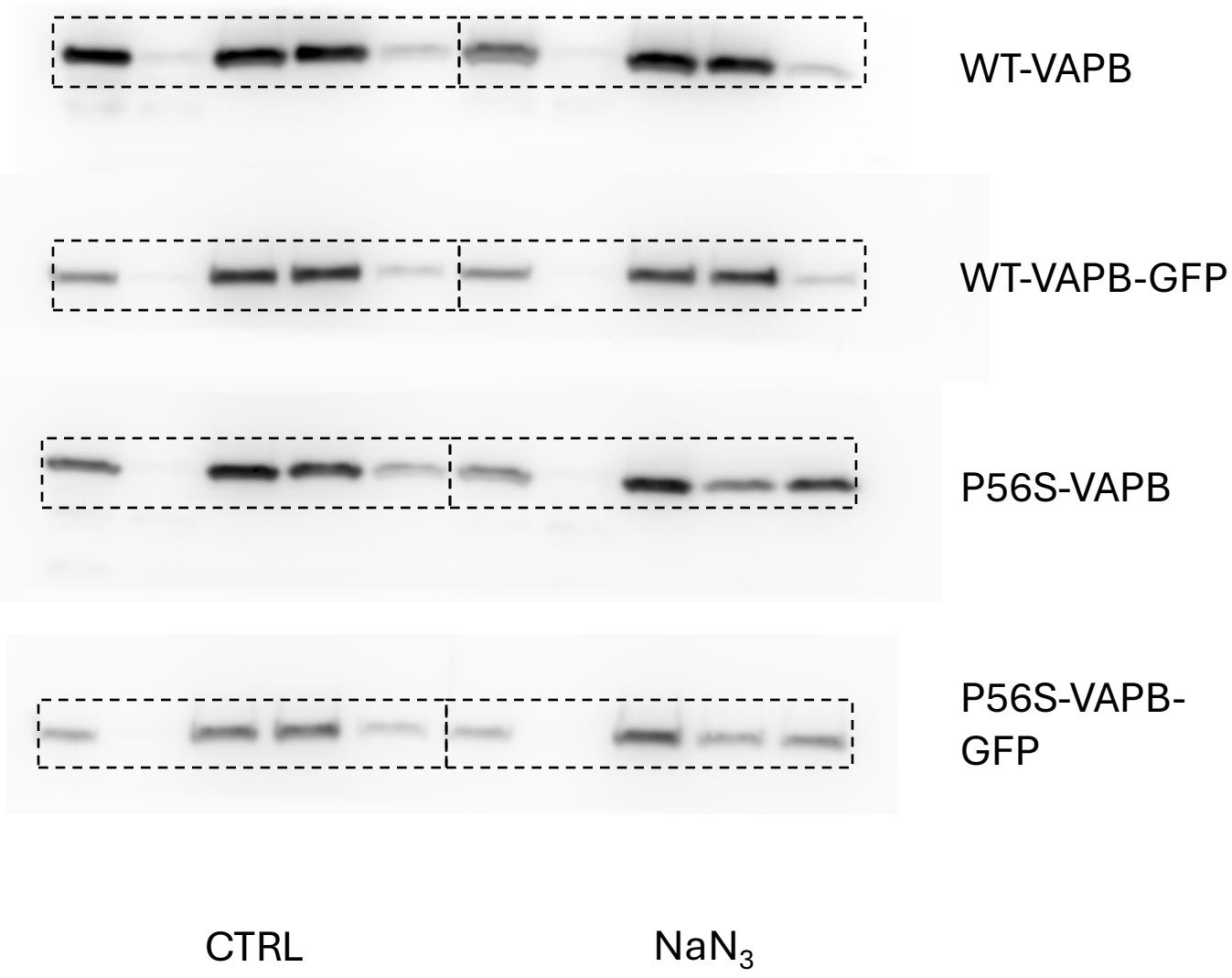

Fig. 2C

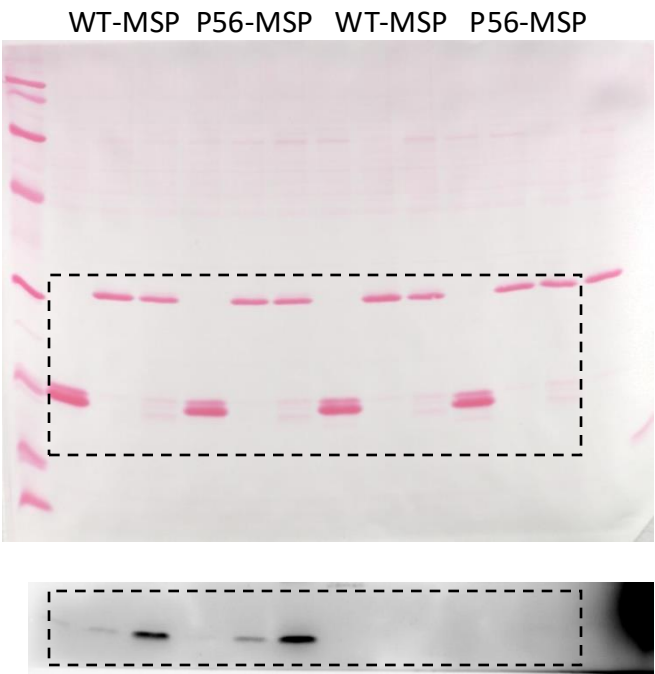

Fig. 2D

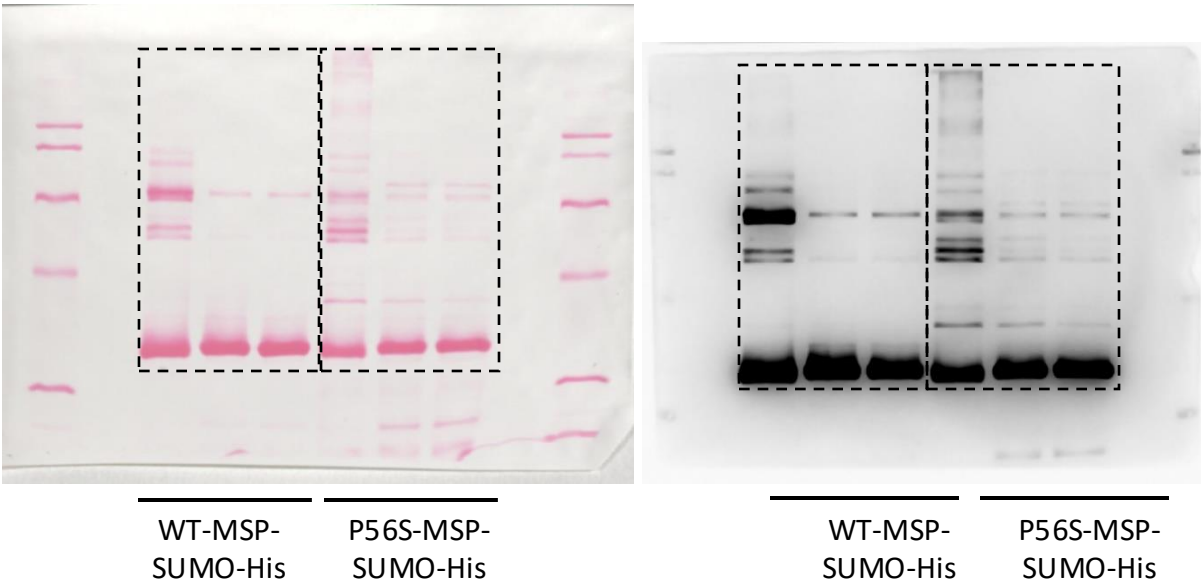

Supplement: Supplementary file 3 [file LSA-2024-02907_SdataF2.2.pdf]

Fig. 4A

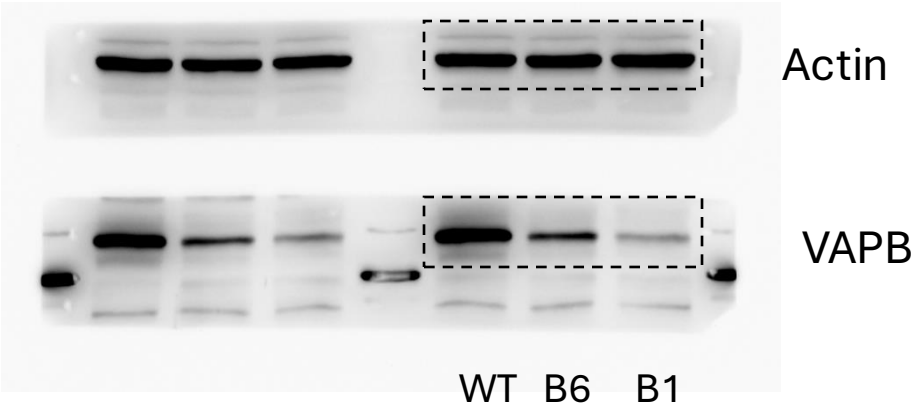

Supplement: Supplementary file 4 [file LSA-2024-02907_SdataF4.1.pdf]
